# Remote ischemic postconditioning protects against crush-induced acute kidney injury via down-regulation of apoptosis and senescence

**DOI:** 10.1007/s00068-022-01910-5

**Published:** 2022-02-28

**Authors:** Heng Jin, Xiaoxi Lin, Ziquan Liu, Jinqiang Wang, Jinxiang Wang, Yan Zhang, Chao Cao, Yanfen Chai, Songtao Shou

**Affiliations:** 1 Department of Emergency Medicine, Tianjin Medical University General Hospital, No. 154, Anshan Road, Heping District, Tianjin, 300050 China; 2 Institute of Disaster Medicine, University of Tianjin, No. 92, Weijin Road, Nankai District, Tianjin, 300072 China; 3 The People’s Hospital of XuChang, No. 1366, Jian’an Street, Xuchang, 461099 Henan Province China

**Keywords:** Crush injury, Ischemic postconditioning, Acute kidney injury, Senescence, Apoptosis

## Abstract

**Background:**

Acute renal failure due to crush syndrome is one of the leading causes of death in disasters. Ischemic Postconditioning (IPC) is a potentially effective strategy to protect against ischemic reperfusion injury, but a few studies noted its protective effect in crush induced acute kidney injury (AKI). Hence, this study investigated the optimal IPC strategy to prevent crush induced AKI and reveal related cellular mechanisms.

**Methods:**

The right lower extremities of rabbits were constantly compressed for 8 h and then performed five cycles of clamping and releasing the femoral artery and vein before depression using a clip. In terms of the duration of clamping and releasing, the animals were randomly divided into 5 groups, Control, IPC-5sec, IPC-30sec, IPC-1min, and IPC-5min groups; 6 rabbits for each group. Biomarkers of inflammation, renal function, renal tubular injury, and muscular injury, apoptosis, and cellular senescence in kidney were detected.

**Results:**

Six hours after decompression, the levels of Serum Creatine (SCr), Blood Urea Nitrogen (BUN), K+, and Interleukin-6 (IL-6) in IPC-1min and IPC-5min groups were lower than Control, with a statistically significant difference. The morphological study of Periodic Acid-Schiff (PAS) staining demonstrated that 6 h after decompression, IPC-1min can attenuate renal tubular damage renal tubule. Meanwhile, the level of Neutrophil Gelatinase-Associated Lipocalin (NGAL) in circulation in the IPC-30sec, IPC-1min, and IPC-5min groups was significantly decreased compared with the Control group, 2 h after decompression. On the other hand, the levels of serum Creatine Kinase (CK) and Myoglobin (Mb), and the morphological change of muscular damage detected by hematoxylin and eosin (H&E) staining in IPC-1min-treated group were significantly lower than Control group 6 hours after decompression. Further results of the cellular mechanism showed that the apoptotic markers of Terminal deoxynucleotidyl Transferase-mediated dUTP Nick End Labeling (TUNEL) and Caspase3 and the cell senescent markers of senescence-associated β-galactosidase (SA-β-Gal) and nuclear LAMNB1 have changed significantly in the IPC-1min group, compared with the control group.

**Conclusions:**

Performing 5 cycles of 1-min IPC would be a convenient, time-saving, and effective method to prevent crush-induced AKI by attenuating the release of nephrotoxic substances after decompression and downregulation of the expression of apoptosis and cellular senescence biomarkers.

## Introduction

Crush-induced acute kidney failure is the second leading cause of high mortality and disability in natural disaster events like earthquakes and major trauma [1], which subsequently increases the physical, psychological, and economic burden [2, 3]. The kidney is the most commonly involved organ in crush syndrome. After decompression, the damaged muscular cells rapidly release nephrotoxic breakdown products such as myoglobin, potassium, and urate into the circulation. Furthermore, it leads to urinary cast formation that causes renal tubule obstruction, proximal tubular necrosis and sequential acute renal failure [4]. Although fluid resuscitation and blood purification are the earliest employed methods that are beneficial and effective for crush injury casualties, immediate therapeutic intervention on the disaster scene is still a challenging issue because the above-mentioned measures are usually unavailable [5]. Moreover, patients wait for more than five hours on average before being transferred to a hospital in the aftermath of the disaster [1]. Severe tissue damage develops around 2–4 h after the compression, which becomes irreversible when the duration exceeds six hours. The increase in compression duration leads to a higher incidence of crush syndrome [6]. Therefore, sincere efforts to explore an effective and feasible measure for protecting the renal function in the pre-hospital care system should be undertaken. A study by Zhao et al. indicated that pretreatment with ischemia–reperfusion cycles could reduce the degree of myocardial ischemia–reperfusion injury and subsequently devised the concept of IPC [7]. Sequential literary insights stated that IPC could mitigate the inherent tissue damage in both renal and cerebral ischemic injury models. However, few studies reported the protective effect of remote IPC on renal injury caused by crush syndrome through clamping of femoral vessels. Although it has been suggested that IPC ameliorates acute kidney injury induced by limb ischemia/reperfusion via inhibiting Toll-like receptor 4 (TLR4) and nuclear factor kappa B (NF-κB) signaling in rats [8], more specific mechanisms require further exploration in this regard. In this study, we evaluated different IPC strategies and investigated the potential mechanisms on a crush injury rabbit models.

## Materials and methods

### Animal models and tissue sample

Thirty-six adult Japanese big-ear rabbits, aged 6–8 months, and weighing 2.3 ± 0.2 kg, were procured from the animal center of the Academy of Military Medical Sciences, China [license: SCXK (army), 2016–0004]. They were raised in single cages for three days, with food and water, at room temperature (22–26 °C), with a humidity of 60–65%, before the study initiation. The study groups were deprived of food and water for 12 h before the implementation of the modeling.

Crush-induced AKI modeling was initiated using a digital crush injury device platform described in a previous study [9]. Anesthesia was administered by pentobarbital sodium solution (1%) through the ear marginal vein in a single dose (3 mL/kg) every three hours. The rabbits’ right lower extremities were constantly compressed at 100 kPa for eight hours. IPC was done by clamping the femoral artery and vein for 5 s, 30 s, 1 min, and 5 min for each group, using a clip, respectively. Moreover, five cycles of clamping and releasing were performed for each rabbit before releasing the clip for the corresponding decompression duration. The animals were divided into five different groups of six rabbits each: control (crush injury without IPC), IPC-5 s, IPC-30 s, IPC-1 min, and IPC-5 min. All animals were sacrificed on the 7th day after decompression. Once the rabbits were anesthetized, the abdomen was dissected, followed by the extraction of kidney tissue. Furthermore, the extruded skin was removed to recover the compressed muscle tissue, which was fixed with paraformaldehyde, embedded in OCT, and frozen at − 80 °C.

### Serum measurements

Ear blood samples were collected at 0, 2, 6, and 12 h after the decompression. The levels of serum creatinine, blood urea nitrogen (BUN), K^+^, and creatine kinase (CK) were measured using a fully automatic biochemical analyzer (JH-6020, CHA), whereas the neutrophil gelatinase-associated lipocalin (NGAL), interleukin-6 (IL-6), and myoglobin (Mb) levels were measured using ELISA kits (Abcam, USA).

### Histopathological analysis

Paraffinized kidney sections (4 µm thick) and the muscle sections were utilized for Periodic acid–Schiff (PAS) and hematoxylin and eosin (H&E) staining by standard laboratory protocols, respectively. The severity of tubular injury [10] and inflammatory infiltrate scores in skeletal muscles [11] were blindly assessed by a pathologist using previously described assessment criteria.

### Immunofluorescence staining

Frozen kidney sections (10 µm thick) were thawed for 30 min at room temperature and sequentially incubated with 0.1% Triton™ X-100 solution for 20 min, 0.1% sodium borohydride solution for 30 min, and a blocking solution (10% goat serum, 0.1% bovine serum albumin in PBS) for 1 h at room temperature. Furthermore, tissues were incubated with primary antibody at 4 °C overnight and then with fluorescently labeled secondary antibody for 1 h at room temperature. Consequently, the sections were mounted using DAPI fluorescent stain. The cleaved Caspase3 (AB3623MI) and LAMNB1 (ab16048) antibodies were taken from Thermo Fisher Scientific and Abcam, respectively. Images were acquired using a Zeiss LSM 710 confocal microscope.

### Senescence-associated β-galactosidase (SA-β-Gal) activity

Kidneys were fixed in PFA (4%) and incubated in sucrose (18%) as previously described. Frozen kidney sections (10 µm thick) were air-dried for 20 min followed by sequential incubation at 37 °C for 12–16 h in fresh SA-β-Gal staining solution. Images were acquired using a bright-field microscope while the percentage of integrated density was calculated by the gray value of the positive area to the whole section using ImageJ software as described below.

### TUNEL assay

Tissues were collected after perfusion with PBS and then embedded in an OCT compound. Frozen kidney sections (10 µm thick) were air-dried for 30 min, incubated with 4% PFA for 20 min at room temperature, permeabilized with 0.1% Triton X-100 in 0.1% sodium citrate solution, and stained according to the quick protocol of the DeadEnd™ Fluorometric TUNEL System (Promega). Images were acquired using a Zeiss Axioplan 2 deconvolution microscope, while the percentage of TUNEL positive cells was calculated by the number of positive cells to the cell nucleus.

### Digital image analysis

The stained kidney sections were used for digital image quantification of each respective signal. For each region, 10 consecutive × 200 optical fields per section were selected for measurement. Signals were quantified by digital treatment of microscopy images using the ImageJ software. The quantification of positive cells was done as follows. A minimum of two good sections on each image was selected for the quantification, followed by subsequent screenshots that converted a muscle section image into a grayscale version. Grayscale was considered proportional to the degree of dyeing. Henceforth, the maximum and minimum thresholds for the grayscale of positive cells were determined so that the maximum number of positive cells were counted. The positive cells were marked, while the non-positive cells were stained with two different colors in the same section. Furthermore, the number of positive and non-positive cells procured from different areas of the section were automatically counted (NIH; https://rsb.info.nih.gov/ij/).

### Statistical analysis

All data were presented as mean ± SD. A statistically significant difference (*p* value ≤ 0.05 was considered significant) was calculated using the two-tailed unpaired Student’s *t* test for two groups, or ANOVA and Tukey’s multiple comparisons test for more than two groups, using SPSS 22.0 software (IBM) or Prism 8 (GraphPad Software).

### Antibodies

Polyclonal antibodies derived from rabbit, Caspase-3 (AB3623MI), and LAMNB1 (ab16048) were from Thermo Fisher Scientific and Abcam, respectively. Secondary antibody (NB7156PCP) derived from goat is polyclonal from Thermo Fisher Scientific and Abcam.

## Results

### IPC improved renal function and alleviated inflammation

**Fig. 1:**
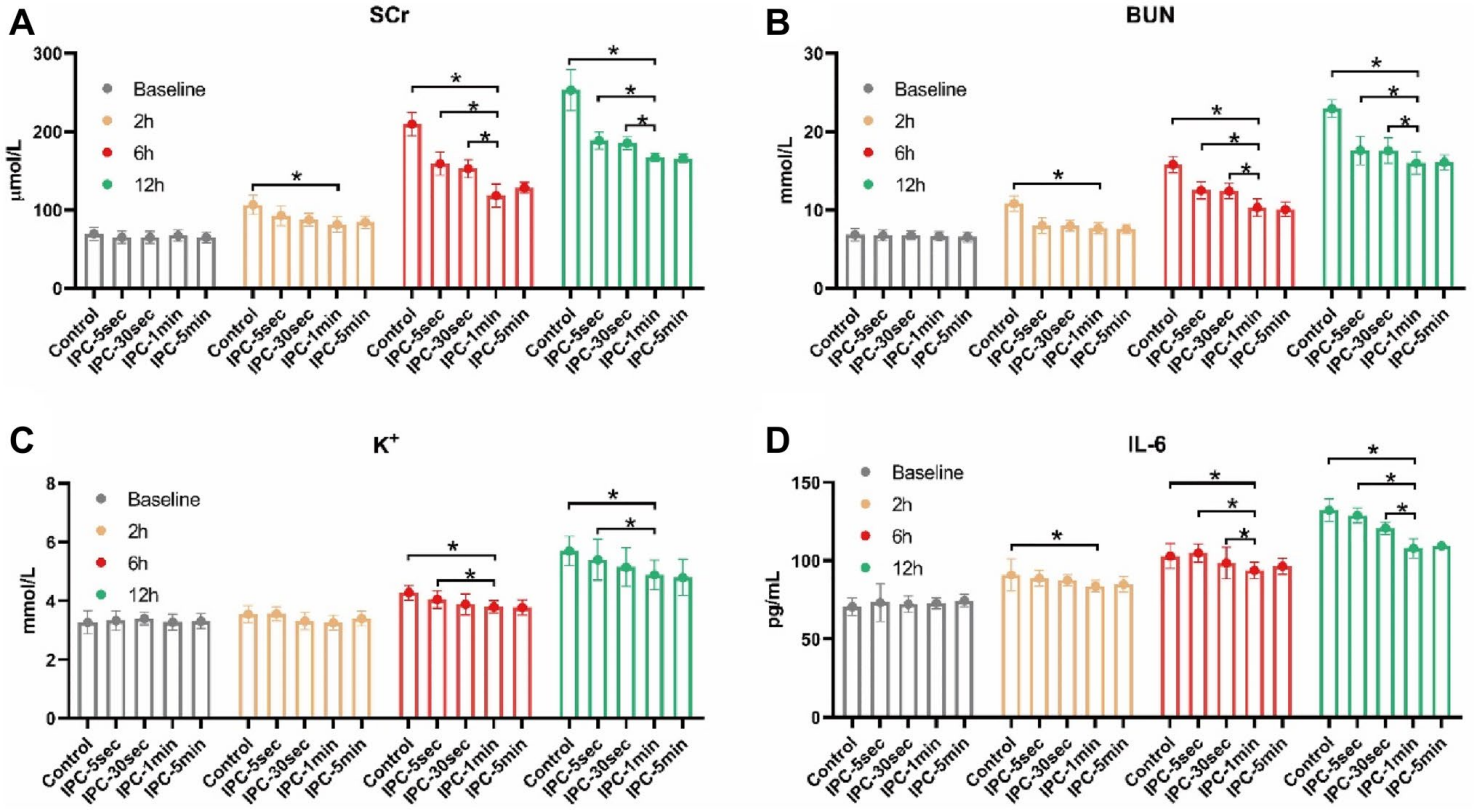
IPC improved renal function and alleviated inflammation. The level of SCr, BUN, K^+^, and IL-6 in IPC-1 min and IPC-5 min groups were lower than control 6 h after decompression with statistically significant difference (1A-D). *p < 0.05

In order to test whether postconditioning improves the renal condition, the levels of SCr, BUN, and K^+^ (with high levels representing the normal renal function), along with the inflammatory biomarker IL-6 in circulation, were measured. The levels of SCr, BUN, K^+^, and IL-6 in IPC-1 min and IPC-5 min groups were lower than in the control group, with a statistically significant difference, six hours after decompression (Fig. 1A–D).

### IPC reduced renal tubular damage

**Fig. 2:**
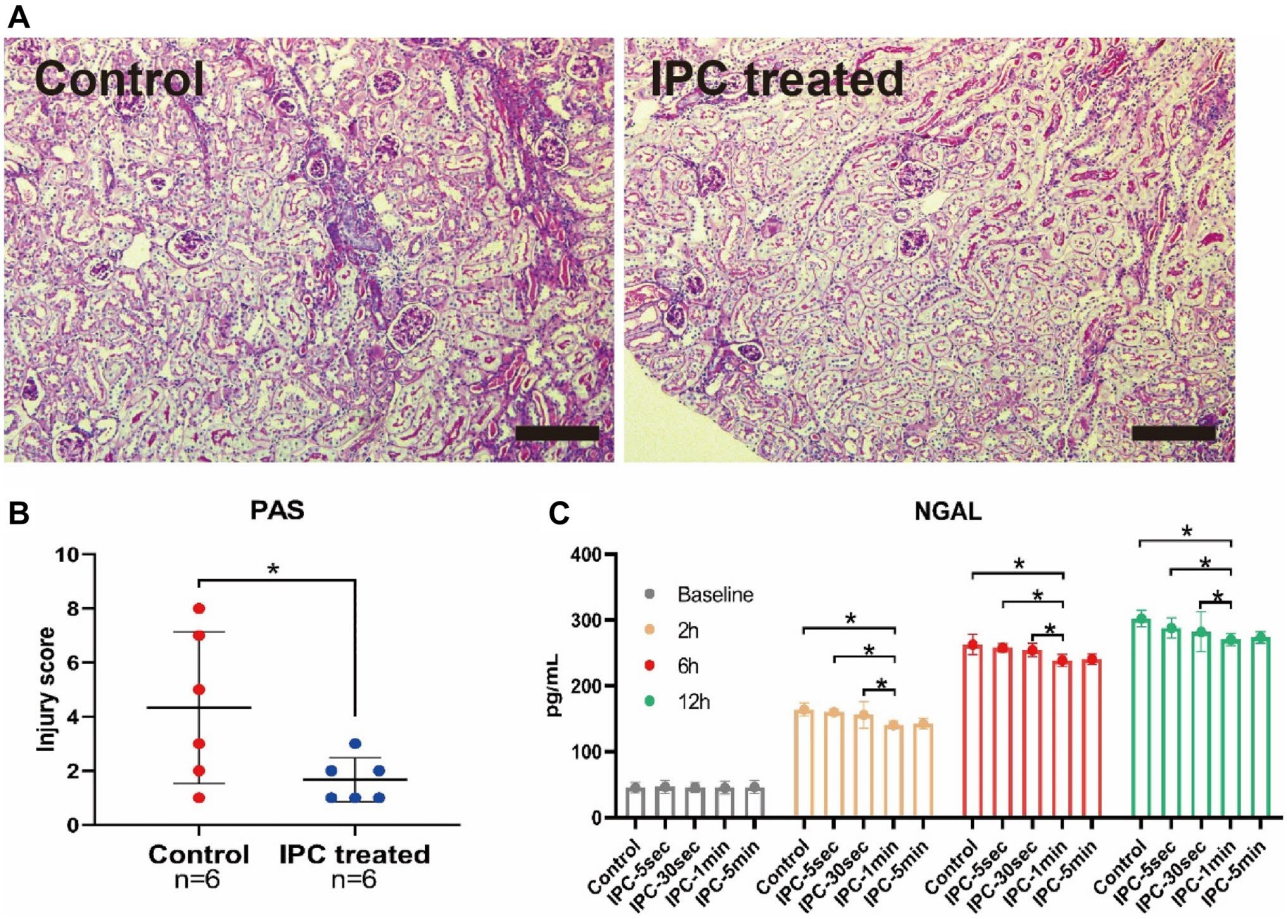
IPC reduced renal tubular damage. Representative images of PAS-stained kidneys at high (lower panels) magnification of IPC-1 min mice compared with the control group (**A**), and tubular injury scores (**B**). The level of NGAL in IPC-30 s, IPC-1 min, and IPC-5 min groups in circulation was significantly decreased when compared with the control group two hours after decompression (**C**). *p < 0.05, scale bar: 200 µm

PAS staining displayed the morphological change in renal tubules while the NGAL level, a biomarker depicting tubular damage was measured. Six hours after decompression, IPC in the IPC-1 min group limited the tubular damage (Fig. 2A), and a relative quantification can be observed in Fig. 2B. The NGAL levels in the IPC-30 s, IPC-1 min, and IPC-5 min groups were significantly decreased when compared with the control group, two hours after decompression (Fig. 2C).

### IPC reduced muscular damage

**Fig. 3:**
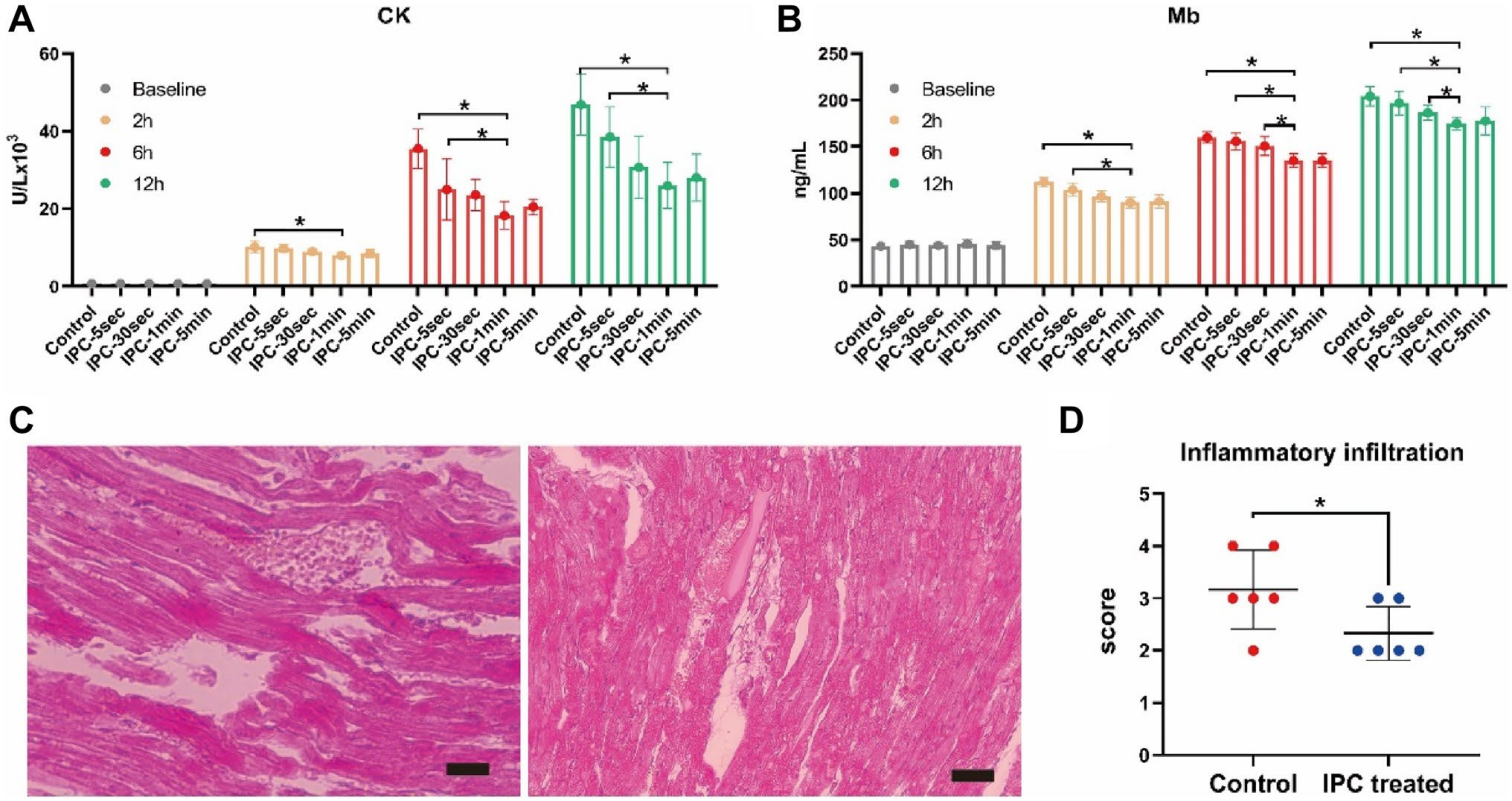
IPC reduced muscular damage. The level of CK and Mb (**A**, **B**) in circulation. H&E stained image of crushed muscular damage after control (left) and IPC-1 min (right) treated six hours after decompression and corresponding quantification (**C**, **D**). *p < 0.05, scale bar: 200 µm

The degree of damage in the renal tubule is directly related to the severity of muscular damage. Therefore, the level of CK and Mb was measured (Fig. 3A, B), which helped in observing the morphological changes. In contrast, the control group revealed apparent muscle fiber fractures, edema, and a large number of neutrophils and lymphocyte infiltration, whereas the IPC-1 min group exhibited only a few inflammatory cells and broken muscle fibers. H&E staining demonstrated that muscular cell swelling and inflammatory infiltration (Fig. 3C, D) were attenuated in the IPC-1 min group.

### IPC downregulated apoptosis and senescence in kidney

**Fig. 4:**
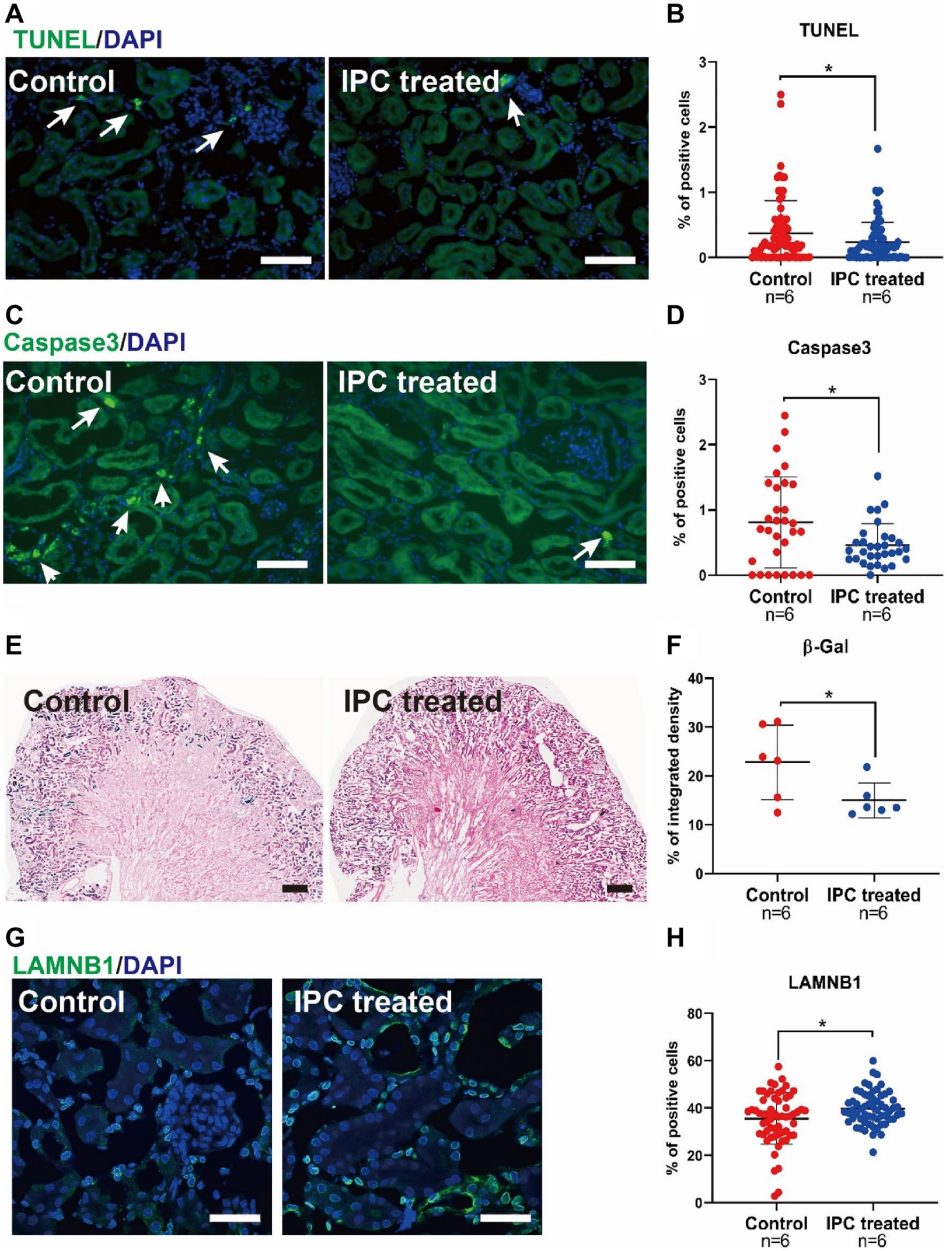
IPC downregulated apoptosis and senescence in kidney. Representative immunofluorescence images of TUNEL and Caspase3 after IPC-1 min treated compared with the control group (**A**–**C**) and corresponding quantification by digital image analysis (**B**–**D**). Representative images of kidneys stained for SA-β-Gal (**E**) and immunofluorescence confocal images for nuclear LAMNB1 (**G**), of IPC-1 min treated group as compared to the control group and corresponding quantification by digital image analysis. *p < 0.05, scale bar: 100 µm (**F**–**H**)

Based on the above results, it was found that IPC-1 min was the optimal strategy, which could be protective against renal tubule damage in a time-saving and effective way. Further exploration of the cellular mechanisms revealed that the IPC reduced the apoptosis, senescence as well as the positive percentage of TUNEL and Caspase3 (Fig. 4A–D), with a decrease in SA-β-Gal activity (Fig. 4E, F) and an abundance of nuclear LAMNB1 (Fig. 4G, H).

## Discussion

Zhao et al. devised the concept of Ischemic Postconditioning (IPC) after the demonstration of the protective effect of ischemia–reperfusion in alleviating myocardial ischemic injury by IPC [7]. IPC has varied potential working mechanisms like the reduction in the oxidative burst (rapid release of reactive oxygen species), antioxidant activity modulation, mitochondrial stabilization, and mitigation of inflammatory cell accumulation and consequent cascade reaction. It is also characterized by intermittent and sequential interruption of blood flow in the early stage of reperfusion. The protective effect of IPC had been reported in a variety of injury models, such as skeletal muscle [12], liver [13], and cerebral ischemia–reperfusion injuries [14]. Additionally, previous studies have demonstrated that different IPC strategies for heart ischemic injury, based on the time of ischemia, reperfusion, and the frequency of cycles, might give rise to totally different outcomes [15]. It was also suggested that IPC can directly protect against ischemia–reperfusion injury in the kidney [16]. However, few studies have focused on the effect of remote IPC protection on crush-induced AKI. Our study utilized four different time frames for investigating an appropriate IPC strategy: 5 s, 30 s, 1 min, and 5 min. The results demonstrated that either 1-min IPC or 5-min IPC was sufficient enough to alleviate renal and muscular histological injury and protect renal function. Since the delay in getting effective treatment is eight hours post the crush injury in an earthquake event [17], the observation period was set from 0 to 12 h. Our study results displayed that 1-min IPC strategy showed an apparent protective effect against kidney and muscular damage as compared to other groups starting from six hours after IPC initiation. These findings suggest that five cycles of 1-min IPC would be a convenient, time-saving and effective method for protecting tissues like kidney and muscle in Crush syndrome (CS).

Many studies have reported that limb remote IPC attenuated the ischemia–reperfusion injury in the brain, heart, and liver, but only a few have reported the effects on the kidney to date [18]. The potential mechanism of remote IPC is represented by the mitigation of oxidative stress, intracellular calcium overload, inflammatory response generation, and eventual cell death [19]. It was shown that IPC ameliorated AKI induced by limb ischemia/reperfusion via inhibiting TLR4 and NF-κB signaling in rats [8]. A meta-analysis by Liu et al. also supported the theory that remote ischemic preconditioning would effectively prevent AKI in patients after cardiac surgery [20]. However, the culminating events in the kidney after IPC are still ambiguous, especially in a crush syndrome model, which varies from typical ischemia–reperfusion injury models. In this study, we investigated the protective effect of remote IPC on the kidney through compressing and releasing limbs. The results demonstrated an improvement in the severity of inflammation in circulation, renal function, and innate kidney and muscle damage. It was evident that IPC and reperfusion cycles after decompression might slow down the release of inflammatory molecules and nephrotoxic substances, causing a pretreatment of the renal tubular cells; thus, the kidney would be able to endure sequential severe insults after complete limb decompression.

Apoptosis (programmed cell death) is one of the key phenomena involved in crush-induced AKI [21]. In order to determine the relation between IPC-induced renal function and apoptotic cell death, the expression of biomarker Caspase3, an executioner of apoptosis, and apoptotic DNA fragmentation was evaluated, which revealed that apoptotic activities decreased in IPC treated groups. This finding suggested that direct apoptotic intervention might become a potential therapeutic intervention for preventing acute renal failure after crushing injuries. On the other hand, recent studies exhibited that cellular senescence, often characterized by apoptotic resistance, occurs in the earlier phase of various AKI models [22, 23] and plays a crucial role in the progression of AKI to CKD [24, 25]. However, the evidence of senescence has not been reported in crush-induced AKI. Therefore, our study model investigated the role of senescence which observed the presence of cellular senescence along with a downregulated expression of senescence in IPC treated groups. The study also unearthed a novel finding that IPC-mediated renal protection was via partial downregulation of senescence. However, it was not possible to evaluate the long-term outcomes due to the limited duration of our study.

## Conclusion

Our study investigated four IPC strategies based on the duration of clamping and releasing femoral vessels and revealed that performing five cycles of 1-min IPC might be a convenient, time-saving, and effective method to prevent crush-induced AKI through downregulation of the apoptosis and the cellular senescence in the kidney. Our findings suggested the effectiveness of apoptosis and cell senescence as potential cellular mechanisms in crush-induced AKI, which might provide a novel path for prompt intervention in acute kidney injury owing to crush syndrome. Future Studies are needed to investigate all inherent molecular mechanisms involved in the cell regulation pathways in such fatal injuries.

## Data Availability

The datasets used and/or analyzed during the current study are available from the corresponding author on reasonable request.
